# Stability of blood eosinophils in acute exacerbation of chronic obstructive pulmonary disease and its relationship to clinical outcomes: a prospective cohort study

**DOI:** 10.1186/s12931-021-01888-5

**Published:** 2021-11-24

**Authors:** Yanan Cui, Wenye Zhang, Yiming Ma, Zijie Zhan, Yan Chen

**Affiliations:** 1 Department of Pulmonary and Critical Care Medicine, The Second Xiangya Hospital, Central South University, Changsha, Hunan China; 2 Research Unit of Respiratory Disease, Central South University, Changsha, Hunan China; 3 Diagnosis and Treatment Center of Respiratory Disease, Changsha, Hunan China

**Keywords:** COPD, Exacerbation, Eosinophil, Stability, Mortality

## Abstract

**Background:**

The clinical value of blood eosinophils and their stability in chronic obstructive pulmonary disease (COPD) remains controversial. There are limited studies on association between the stability of blood eosinophils in acute exacerbation of COPD (AECOPD) and clinical outcomes. This study aimed to evaluate the stability of blood eosinophils in hospitalized AECOPD and its relationship to clinical outcomes.

**Methods:**

This prospective observational study recruited patients hospitalized with AECOPD from November 2016 to July 2020. The eligible patients were divided into four groups according to their blood eosinophil counts at admission and discharge: persistently < 300 cells/μl (LL), < 300 cells/μl at admission but ≥ 300 cells/µl at discharge (LH), ≥ 300 cells/μl at admission but < 300 cells/µl at discharge (HL), and persistently ≥ 300 cells/μl (HH). Cox hazard analyses were used to study the association between eosinophil changes and exacerbations or mortality.

**Results:**

In 530 patients included, 90 (17.0%) had a high blood eosinophil count (BEC) ≥ 300 cells/µl at admission but 32 (35.6%) of them showed a decreased BEC at discharge. The proportions and distribution for group LL, LH, HL, and HH were 381 (71.9%), 59 (11.1%), 32 (6.0%), and 58 (10.9%), respectively. During hospitalization, the LH group had a higher C-reactive protein level, higher rate of intensive care unit (ICU) admission, and higher total cost. The length of hospital stay of the LH group was longer compared with group LL, HL, or HH (*P* = 0.002, 0.017, and 0.001, respectively). During a follow-up of 12 months, the HH group was associated with a higher risk of moderate-to-severe exacerbations compared to the LL group (hazard ratio 2.00, 95% confidence interval 1.30–3.08, *P* = 0.002). Eosinophil changes had no significant association with mortality at 12 months. Sensitivity analyses in patients without asthma and without use of systemic corticosteroids prior to admission did not alter the results.

**Conclusions:**

More attention should be paid to the LH group when evaluating the short-term prognosis of AECOPD. A persistently high BEC was a risk factor for long-term exacerbations. Eosinophil changes during hospitalization could help to predict outcomes.

**Supplementary Information:**

The online version contains supplementary material available at 10.1186/s12931-021-01888-5.

## Background

Chronic obstructive pulmonary disease (COPD) is a highly heterogeneous disease and the underlying cellular and molecular mechanisms of COPD remain insufficient understanding. The use of biomarkers could help to stratify management for COPD and improve patient outcomes [1].

The Global Initiative for Chronic Obstructive Lung Disease (GOLD) 2021 recommended blood eosinophil counts as a biomarker helping clinicians estimate the benefits of using inhaled corticosteroids (ICS) [2]. However, current studies have produced conflicting results regarding the ability of blood eosinophils to predict future exacerbation risk [3, 4].

The repeatability of blood eosinophil counts also appears to be controversial. A study including patients from the COPDMAP cohort reported that approximately 70% of blood eosinophil measurements remained stable over 1 year [5], but in the PROMISE-COPD study, blood eosinophil counts presented significant variability throughout the course of COPD [6]. Moreover, scarce data exist regarding the association between the variability of blood eosinophils and outcomes. Data from the CHAIN and BODE cohorts showed that persistently elevated eosinophil counts in stable COPD patients was not a risk factor for COPD exacerbations [7], but a population-based study recently found patients with the highest variability in blood eosinophils more frequently experienced exacerbations [8].

A meta-analysis including observational studies and randomized controlled trials (RCTs) in 2017 reported that, in patients with acute exacerbation of COPD (AECOPD), blood eosinophilia was associated with reduced length of hospital stay but showed no association with risk of exacerbation in 12 months [9]. Another recent meta-analysis including RCTs and quasi-experimental studies also showed that eosinophilic AECOPD had a shorter length of hospital stay [10]. However, in these two reviews, there was no study exploring the stability of blood eosinophils during an AECOPD and its relationship to clinical outcomes. In addition, the value of eosinophils in evaluating the economic burden of AECOPD during hospitalization was less reported.

In the current study, we assessed blood eosinophil counts on admission and discharge in patients hospitalized due to an exacerbation and sought their associations with short-term outcomes including the length of stay and healthcare costs and long-term adverse events to test the hypothesis that eosinophil changes during hospitalization could help to predict clinical outcomes.

## Methods

### Study design and study population

This prospective observational study recruited patients admitted with the primary diagnosis of AECOPD to the Department of Respiratory and Critical Care Medicine, the Second Xiangya Hospital of Central South University, Hunan, China between November 2016 and July 2020. COPD had been previously diagnosed by a respiratory specialist according to the GOLD (including evaluation of symptoms, risk factors, and persistent airflow limitation defined as a post-bronchodilator forced expiratory volume in 1 s/forced vital capacity < 0.70) [11]. Exacerbations were defined as a worsening of respiratory symptoms that required additional therapy [11]. This study was approved by the Ethics Committee of the Second Xiangya Hospital of Central South University (No. ChiCTR-POC-17010431) and conducted in accordance with the Declaration of Helsinki. All patients provided written informed consent in this study.

All the included patients had a minimum of two blood eosinophil measurements. The first measurement time was between 24 h before admission and 24 h after admission. Another test was conducted within 3 days before discharge. Patients were excluded if they had pulmonary embolism, pulmonary hypertension, interstitial pulmonary disease, advanced lung cancer, active pulmonary tuberculosis or bronchiectasis. Those with a history of any comorbidity that could influence blood eosinophil count (BEC) (such as allergic disorder, autoimmune disease, or hematologic disease) were also excluded. A threshold value of 300 cells/µL was used to define blood eosinophilia, since this threshold had previously showed the largest independent effect of ICS on reducing the risk of exacerbation in a systematic review of post-hoc analyses of RCTs [12] and the threshold was also recommended by the GOLD 2021 to guide therapy [2]. To investigate the stability of blood eosinophils during an exacerbation we divided participants into four groups: patients with a low BEC < 300 cells/μl at admission and discharge (LL), patients with a low BEC < 300 cells/μl at admission but a high BEC ≥ 300 cells/µl at discharge (LH), patients with a high BEC ≥ 300 cells/μl at admission but a low BEC < 300 cells/µl at discharge (HL), and patients with a high BEC ≥ 300 cells/μl at admission and discharge (HH).

### Measurements and outcomes

Patient clinical characteristics, including age, sex, body-mass index (BMI), smoking status, pulmonary function, 6-Min walk test, modified Medical Research Council (mMRC) dyspnea grade, COPD Assessment Test (CAT) score, and respiratory failure were recorded upon admission. Exacerbations in the previous year, pre-admission medications, and comorbidities were defined according to the historical clinical records. Blood routine measurements, C-reactive protein (CRP) test, respiratory support use, intensive care unit (ICU) admission, length of hospital stay, and total cost during hospitalization were also obtained. The costs were shown in US dollars using the average exchange rate in the first half of 2021 (one US dollar was equivalent to 6.47 Chinese yuan).

Patients were followed-up for 12 months by phone calls every 3 months or by direct patient interviews upon re-admission. Data on moderate or severe exacerbations and mortality were collected. Moderate exacerbations were defined as the need for antibiotics and/or systemic corticosteroids due to worsening respiratory symptoms, while severe exacerbations required hospitalization or emergency department visits [11].

The primary outcomes were the length of stay in hospital, moderate-to-severe exacerbations and all-cause mortality within 12 months after discharge. Secondary outcomes were the CRP level, ICU admission rate, and the total cost during hospitalization.

### Sensitivity analyses

In order to further eliminate the influence of comorbidity or corticosteroid use to the BEC or outcomes, sensitivity analyses were performed by excluding patients with asthma or receiving systemic corticosteroids prior to admission.

### Statistical analyses

Statistical analyses were performed using the software IBM-SPSS statistics 25. Continuous data were expressed as median (interquartile range, IQR) and mean (standard deviation, SD), and categorical data were described as frequencies (percentage). Comparison of categorical variables were performed using the chi-squared test or Fisher’s exact test. For continuous variables, comparisons were made between groups using the one-way ANOVA, the Mann–Whitney U-test or the t-test as appropriate. The Cox proportional hazards regression models were conducted to evaluate the hazard ratios between eosinophil changes and moderate-to-severe exacerbations or all-cause mortality. The potential confounding factors showing statistical differences between groups in baseline characteristic analyses (age and sex) were adjusted in the models. A two-sided *P*-value < 0.05 was considered statistically significant.

## Results

### Characteristics of the participants

**Fig. 1:**
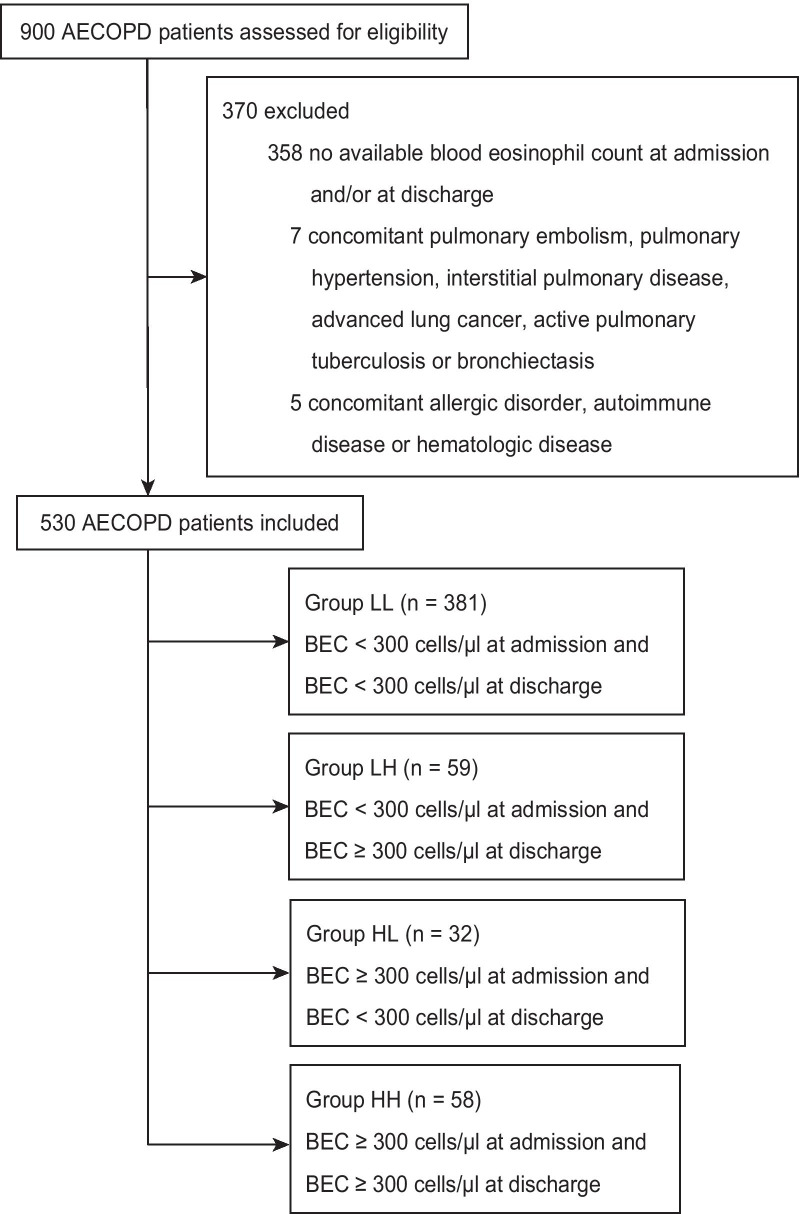
Flowchart of eligible study population. *AECOPD* acute exacerbations of chronic obstructive pulmonary disease, *BEC* blood eosinophil count

Among the 900 patients assessed for eligibility, 530 patients were included and followed up in this study (Fig. 1). At admission, the BEC was < 300 cells/μl in 440 patients (83.0%), of whom 59 (13.4%) were discharged with elevated blood eosinophils. 90 (17.0%) had a high BEC ≥ 300 cells/µl at admission but 32 (35.6%) of them showed a decreased BEC at discharge. The proportions and distribution for group LL, LH, HL, and HH were 381 (71.9%), 59 (11.1%), 32 (6.0%), and 58 (10.9%), respectively. The median age of the study population was 68 years and 91.1% were male.

The baseline characteristics of 530 patients with AECOPD are shown in Table 1. There were no statistical differences among the four groups except for age, sex, and the proportion of patients with asthma. The patients in the LL group were older than those in the HL group (*P* = 0.002) or in the HH group (*P* = 0.020). The proportions of female patients and patients with asthma were significantly higher in the HL group than in the LL group (*P* = 0.010 and 0.001, respectively).

**Table 1:** Clinical characteristics of the study population

Variables	Total cohort (n = 530)	Blood eosinophil count				
		LL (n = 381)	LH (n = 59)	HL (n = 32)	HH (n = 58)	*P*-value
Age (years)	68 (63–76)	69 (64–77)	67 (61–75)	66 (57–70)^a^	66 (61–72)^a^	0.002
Male	483 (91.1%)	354 (92.9%)	52 (88.1%)	25 (78.1%)^a^	52 (89.7%)	0.028
Body-mass index (kg/m^2^)	21.2 (18.7–24.1)	21.1 (18.5–24.2)	21.8 (19.1–24.2)	21.5 (20.1–23.4)	20.6 (18.3–23.5)	0.770
Smoking status^d^						0.127
Non-smoker	70 (13.3%)	43 (11.4%)	9 (15.3%)	9 (28.1%)	9 (15.5%)	
Ex-smoker	304 (57.8%)	218 (57.8%)	36 (61.0%)	14 (43.8%)	36 (62.1%)	
Current smoker	152 (28.9%)	116 (30.8%)	14 (23.7%)	9 (28.1%)	13 (22.4%)	
Post-bronchodilator FEV_1_% predicted	32.1 (22.9–43.0)	33.3 (23.0–43.3)	34.1 (22.1–47.2)	31.4 (22.5–36.5)	30.5 (22.3–38.5)	0.337
Post-bronchodilator FEV_1_/FVC (%)	37.8 (31.0–48.3)	38.0 (31.1–50.0)	34.7 (27.6–48.9)	38.2 (32.2–46.6)	34.4 (31.2–41.0)	0.186
6-Min walk test (m)	127.0 (10.0–300.0)	120.0 (10.0–307.0)	105.0 (0.0–256.8)	192.0 (50.0–296.3)	180.0 (50.0–304.5)	0.692
mMRC	3 (2–4)	3 (2–4)	3 (2–4)	3 (3–4)	3 (3–4)	0.406
CAT	24 (18–29)	24 (18–28)	22 (19–30)	26 (21–30)	25 (18–30)	0.779
Exacerbations in the previous year	2 (1–3)	2 (1–3)	2 (1–3)	2 (1–3)	2 (1–4)	0.238
Pre-admission medication^e^						
LAMA	238 (45.3%)	169 (44.5%)	28 (50.0%)	14 (43.8%)	27 (47.4%)	0.872
LABA	308 (58.7%)	219 (57.6%)	33 (58.9%)	19 (59.4%)	37 (64.9%)	0.784
ICS	302 (57.5%)	216 (56.8%)	33 (58.9%)	18 (56.3%)	35 (61.4%)	0.920
Systemic corticosteroids	54 (10.3%)	37 (9.7%)	8 (14.3%)	2 (6.3%)	7 (12.3%)	0.588
Co-morbidity						
Pneumonia	101 (19.1%)	72 (18.9%)	14 (23.7%)	5 (15.6%)	10 (17.2%)	0.767
Asthma	51 (9.6%)	30 (7.9%)	8 (13.6%)	9 (28.1%)^a^	4 (6.9%)^c^	0.002
Hypertension	200 (37.7%)	145 (38.1%)	22 (37.3%)	11 (34.4%)	22 (37.9%)	0.985
Ischaemic heart disease	90 (17.0%)	67 (17.6%)	8 (13.6%)	2 (6.3%)	13 (22.4%)	0.219
Diabetes	72 (13.6%)	55 (14.4%)	5 (8.5%)	4 (12.5%)	8 (13.8%)	0.671
Cerebrovascular accident	36 (6.8%)	31 (8.1%)	4 (6.8%)	1 (3.1%)	0 (0.0%)	0.078
Respiratory failure	232 (43.8%)	174 (45.7%)	24 (40.7%)	10 (31.3%)	24 (41.4%)	0.401
Systemic corticosteroids during admission	438 (82.6%)	316 (82.9%)	47 (79.7%)	28 (87.5%)	47 (81.0%)	0.806
Respiratory support during admission						
Oxygen therapy	370 (69.8%)	264 (69.3%)	41 (69.5%)	21 (65.6%)	44 (75.9%)	0.727
NIV	238 (44.9%)	179 (47.0%)	26 (44.1%)	13 (40.6%)	20 (34.5%)	0.325
IMV	10 (1.9%)	5 (1.3%)	4 (6.8%)^a^	1 (3.1%)	0 (0.0%)	0.033
ICU during admission	71 (13.4%)	53 (13.9%)	15 (25.4%)^a^	1 (3.1%)^b^	2 (3.4%)^a, b^	0.002

Data are presented as n (%) or median (IQR). ^a^Compared with the LL group, P < 0.05. ^b^Compared with the LH group, P < 0.05. ^c^Compared with the HL group, P < 0.05. ^d^Smoking status has 4 missing values. ^e^Pre-admission medication has 5 missing values

*FEV*_*1*_ forced expiratory volume in 1 s, *FVC* forced vital capacity, *mMRC* modified Medical Research Council, *CAT* COPD Assessment Test, *LAMA* long-acting muscarinic receptor antagonist, *LABA* long-acting beta-adrenoceptor agonist, *ICS* inhaled corticosteroids, *NIV* noninvasive ventilation, *IMV* invasive mechanical ventilation, *ICU* intensive care unit

**Fig. 2:**
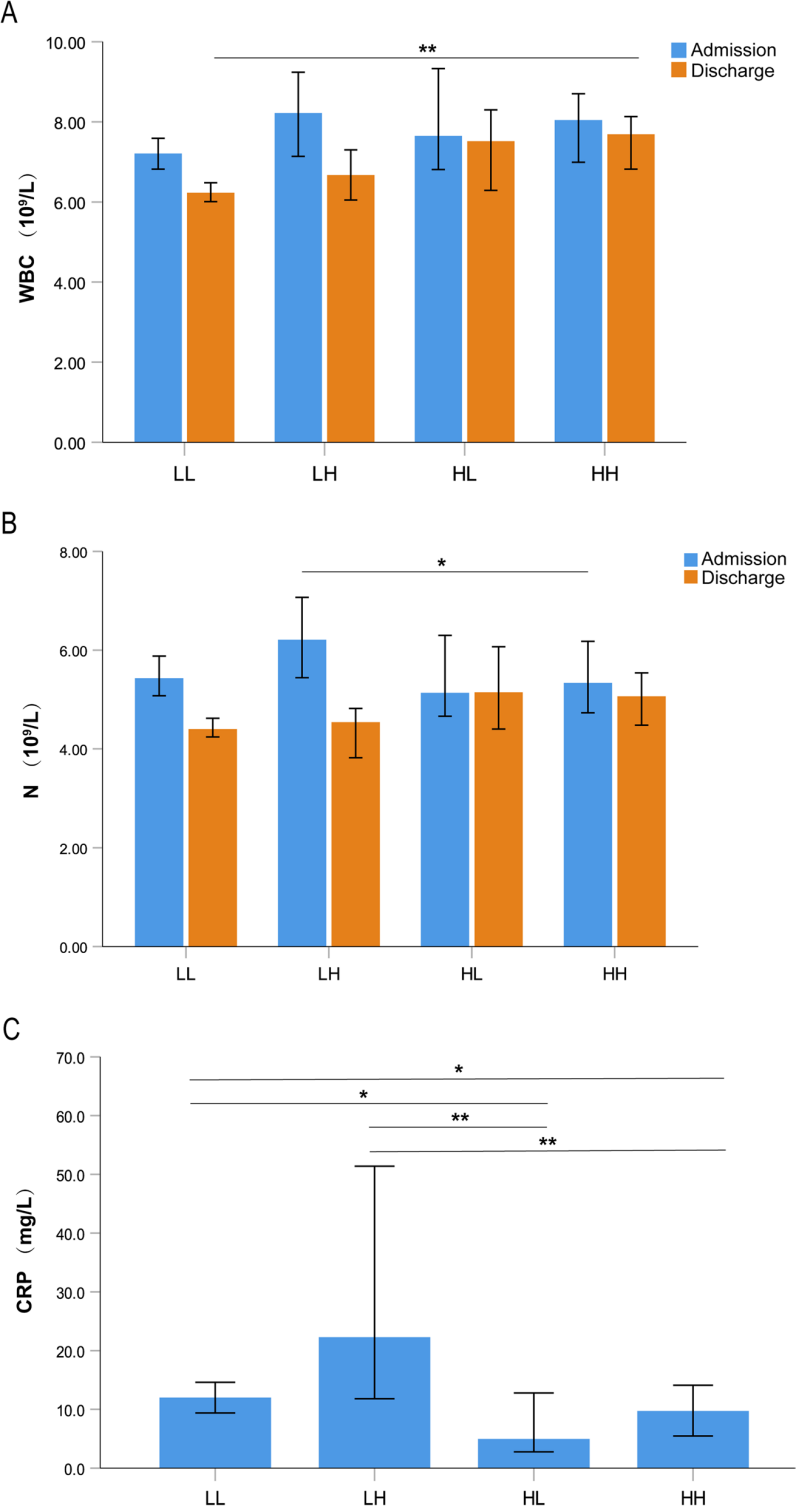
Laboratory data including WBC (**A**), N (**B**), and CRP (**C**) according to blood eosinophil group. Data are presented as median. Error bars show 95% confidence interval. Statistically significant differences between groups are indicated as ^*^P ≤ 0.05 and ^**^P ≤ 0.01. *WBC* white blood cells, *N* neutrophils, *CRP* C-reactive protein

**Fig. 3:**
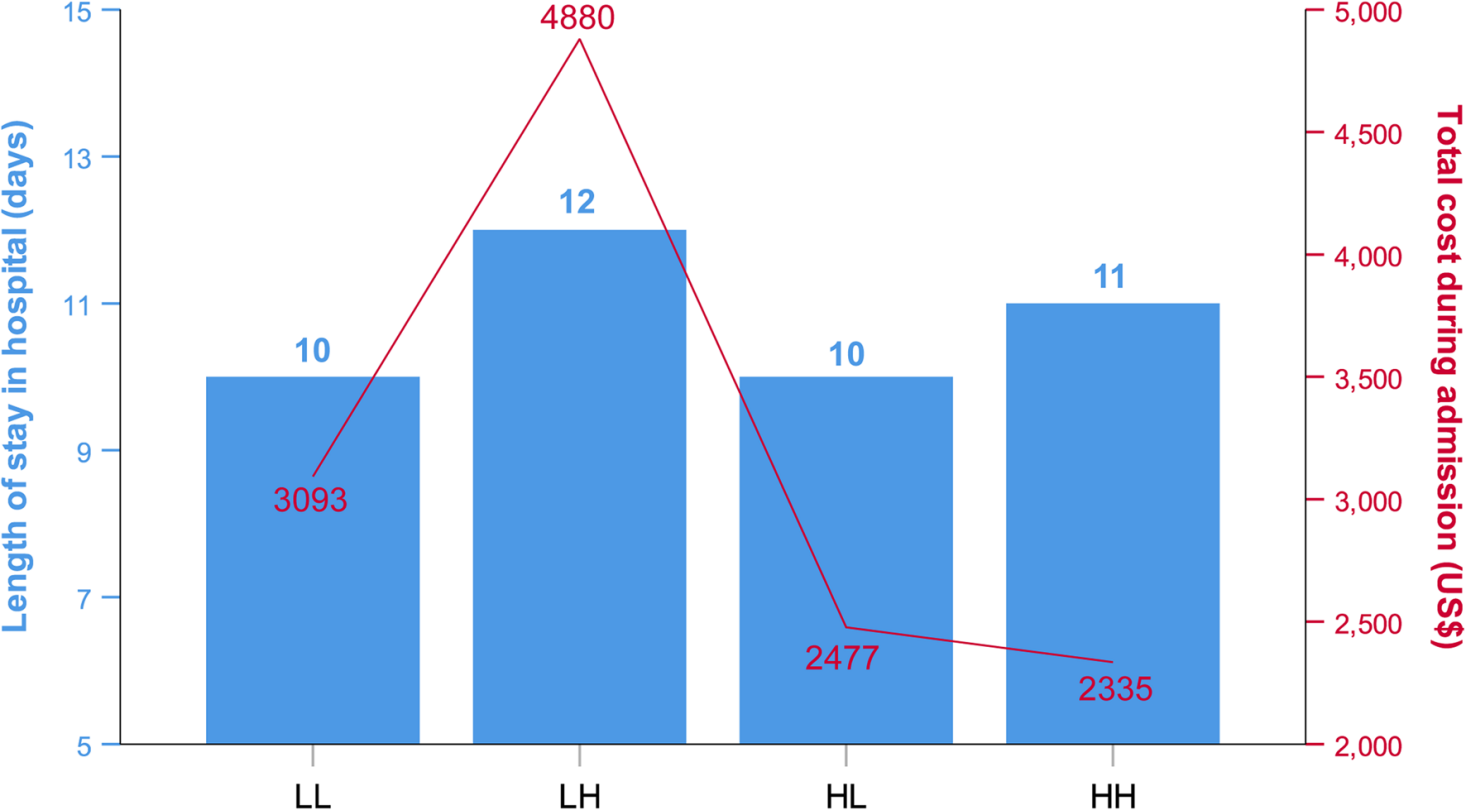
Length of stay in hospital^a^ and total cost during admission^b^ according to blood eosinophil group. ^a^Data are presented as median. ^b^Data are presented as mean

During hospitalization, the LH group had the greatest changes in counts of white blood cells (WBC) and neutrophils (Fig. 2A, B). The median CRP was higher in patients with lower eosinophil counts at admission and reached the highest in group LH (Fig. 2C). In addition, the LH group had the highest rates of invasive mechanical ventilation (IMV) use and ICU admission (Table 1). The median time in hospital of the LH group was significantly longer compared with group LL, HL, or HH (*P* = 0.002, 0.017, and 0.001, respectively), whereas no statistical difference was found between these three groups (Fig. 3). The total cost during hospitalization was highest in the LH group (all *P* values < 0.001 compared with the other three groups), but the differences were not statistically significant among group LL, HL, and HH (Fig. 3).

### Longitudinal analyses

A total of 349 patients (65.8%) of 530 had follow-up data within 12 months after discharge. There was no significant difference in clinical characteristics including the BEC among subjects with and without follow-up data (Additional file 1: Table S1).

**Fig. 4:**
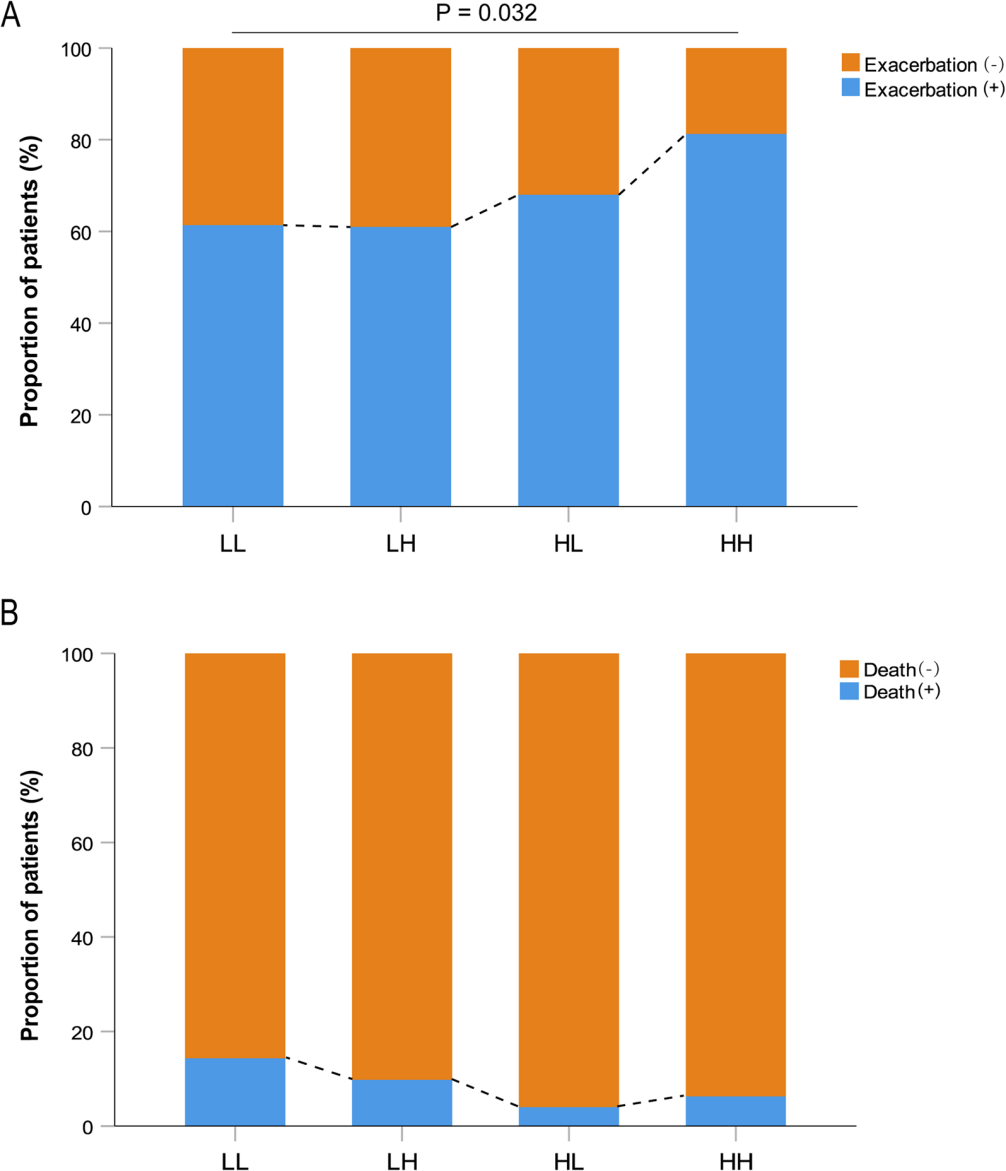
Frequency of moderate-to-severe exacerbations **A** and death **B** according to blood eosinophil group during follow-up

**Fig. 5:**
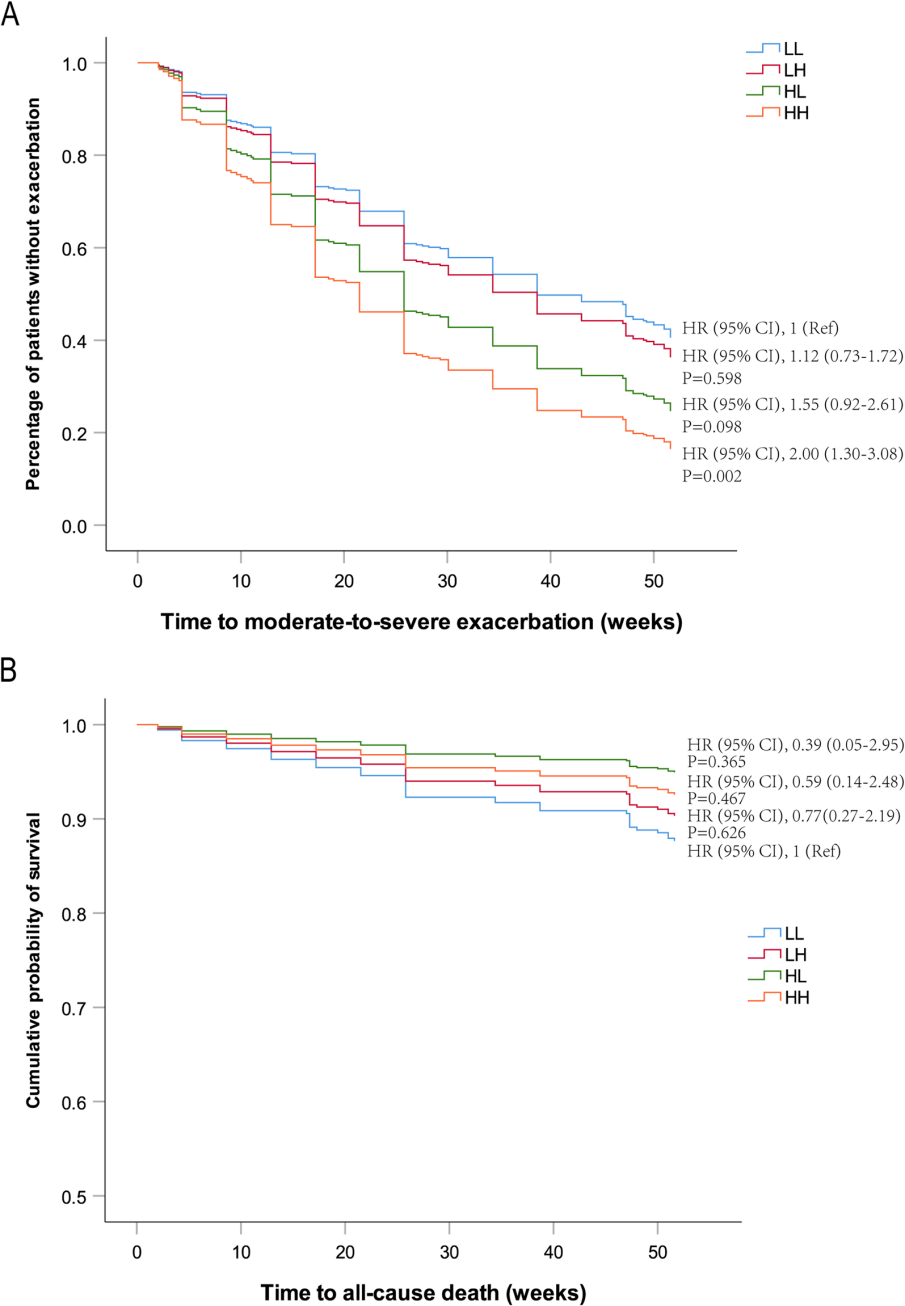
Survival analyses according to blood eosinophil group using Cox proportional hazards model. Time to moderate-to-severe exacerbation **A** or all-cause death (**B**)

Moderate-to-severe exacerbations occurred in 222 (63.6%) patients during follow-up. The proportions of patients with one or more moderate-to-severe exacerbations and the distribution for group LL, LH, HL, and HH were 154 (61.4%), 25 (61.0%), 17 (68.0%), and 26 (81.3%), respectively, with significant difference between group LL and HH (*P* = 0.032; Fig. 4). The time to the first exacerbation shortened gradually from group LL to HH (Fig. 5). Cox regression analyses showed, for the risk of exacerbations, only the difference between group LL and HH was statistically significant and the HH group was associated with a higher risk compared to the LL group (HR = 2.00, 95% CI, 1.30–3.08, *P* = 0.002; Fig. 5).

There were 43 (12.3%) deaths, of which 36 (14.3%) were in the LL group, 4 (9.8%) were in the LH group, 1 (4.0%) was in the HL group, and 2 (6.3%) were in the HH group (*P* = 0.355). The all-cause mortality tended to decrease gradually from group LL to HH (Fig. 4). Compared to the LL group, time to death showed no significant difference in group LH (HR = 0.77, 95% CI, 0.27–2.19, *P* = 0.626), HL (HR = 0.39, 95% CI, 0.05–2.95, *P* = 0.365), and HH (HR = 0.59, 95% CI, 0.14–2.48, *P* = 0.467) (Fig. 5).

### Sensitivity analyses

Sensitivity analyses were conducted in 434 patients without asthma and without use of systemic corticosteroids prior to admission (Additional file 1: Table S2). Similarly, the LH group had the longest length of hospital stay and the highest total cost during hospitalization. During follow-up, the HH group had the highest risk of moderate-to-severe exacerbations but appeared to have a lower risk of death (Additional file 1: Table S3).

## Discussion

To the best of our knowledge, this has been the first prospective observational study with relatively recent recruitment of patients evaluating the stability of blood eosinophils during an acute exacerbation and its relationship to clinical outcomes. This study yielded several important findings. First, during an exacerbation requiring hospital admission, more than one-third of patients admitted with a BEC ≥ 300 cells/µl were discharged with a BEC < 300 cells/μl. Second, a low BEC at admission but high at discharge was associated with a higher CRP level, higher rate of ICU admission, longer length of stay, and higher total cost during hospitalization. Third, the value of eosinophils in predicting future exacerbations was meaningful only in patients with a persistently low or high BEC. A persistently high BEC during admission was associated with a higher risk of exacerbations, while a persistently low BEC might be related to poor survival.

In our study, a BEC ≥ 300 cells/µl at admission was found in 17% of patients hospitalized due to an exacerbation, similar to the studies in Germany [13] and Spain [14]. 10.9% of patients included in this study had a persistently high BEC. Few studies reported the stability of eosinophils in exacerbated COPD. Schumann et al. recruiting 210 patients in stable COPD with 312 hospitalized visits for severe AECOPD during follow-up reported that the concordance (> 300 cells/µl) was only 5% during severe exacerbations [6]. Notably, the data analyzed in this study were based on multiple visits over an average of 4 years and the repeatability tended to progressively decline with the increase of follow-up time [15]. Recently, a study in China including 241 AECOPD patients with at least three blood eosinophil counts showed that 17.0% had a high BEC at almost each hospitalization during the study period, but the threshold value of blood eosinophils was 150 cells/μL in this study [16]. No previous study investigated the changes of blood eosinophils during an AECOPD.

For eosinophils at admission and discharge, we found a discordance of 35.6% in patients admitted with a BEC ≥ 300 cells/µl, far exceeding the degree of inconsistency in patients with a low BEC at admission. This result is in line with those of recent studies. Southworth et al. proposed that a greater variability was found at higher blood eosinophil counts and the greatest variation was observed in patients with a BEC ≥ 300 cells/µl [17]. A retrospective cohort analysis in UK and US COPD populations found that patients with BEC < 150 cells/µl had the highest stability [18].

In this study, we identified a group of patients whose eosinophil counts were low at admission but high at discharge showing specific clinical characteristics. These patients had a stronger general inflammatory reaction compared with other patients, based on their levels and counts of CRP, WBC, and neutrophils. Although some studies suggested that blood eosinophil counts negatively correlated with CRP levels and neutrophil counts [13, 19, 20], few scholars have ever paid attention to the differences between patients with a persistently low BEC and those with increasing eosinophils later. The differences of CRP between group LH and group HL or HH were more significant than that between group LL and group HL or HH. A significantly increased neutrophil count at admission was observed only in group LH rather than in group LL.

In addition, most previous researches explored the relationship between blood eosinophils and length of hospital stay according to the level of a single BEC during AECOPD. Some studies showed that higher eosinophil levels were associated with shorter length of stays [13, 21, 22], but other studies found no relationship [14, 23] or even reported a longer hospital stay [24]. The two systematic reviews mentioned above both proposed that non-eosinophilic AECOPD had a longer length of hospital stay [9, 10]. In our study, patients admitted with a low BEC but increasing at discharge had the longest hospital stay and no difference was found between patients with a persistently low BEC and patients with a persistently high BEC. Generally, only the BEC at admission was used for analyses in previous studies. The LL group was not separated from those subjects with a low BEC at admission, which could influence the accuracy of the results. Intriguingly, similar results were observed in the analysis of hospital expenses. The finding that lower eosinophil counts were associated with higher economic burden of hospitalization was consistent with our recent study using real-world data in China [23]. The higher total cost in the LH group might be related to their stronger general inflammatory reaction, higher rate of ICU admission and longer hospital stay.

The value of blood eosinophils during an exacerbation in predicting future exacerbation risk remains controversial. In an observational prospective cohort study conducted in 152 patients, no association was found between eosinophilia (≥ 3% of total WBC and/or ≥ 300 cells/µl) and the time to the next moderate-to-severe exacerbation [25]. A retrospective cohort study grouping 508 patients as ≤ 100, 100–300, and ≥ 300 eosinophils/µL also proposed that eosinophil count was not associated with exacerbation within 1 year after discharge [26], whereas another retrospective study including 2445 patients showed that a BEC ≥ 300 cells/µl was associated with increased risk of COPD-related readmission at 12 months [27]. In the meta-analysis conducted by Ho et al. eosinophilic AECOPD showed no association with risk of exacerbation in 12 months [9]. However, only a single BEC was used in these studies to evaluate prognosis. Using two measurements at different time points, we further revealed that only patients with a persistently high BEC during admission had a higher risk of moderate-to-severe exacerbations and patients admitted with a high BEC but decreasing at discharge did not show an increased risk of exacerbation. In previous studies, eosinophilic AECOPD patients included those in the HL group, thereby limiting the possibility of finding the potential significant associations. Other studies investigating the variability of BEC and exacerbation risk were carried out mainly in stable COPD patients [7, 8, 28].

The current study found that patients with a persistently low BEC tended to have poor survival, although no significant difference was observed. In fact, the study in Chinese patients with at least three blood eosinophils measured from different hospitalizations has reported that patients with predominantly increased eosinophils (≥ 150 cells/µl) had a lower risk of all-cause mortality compared to those with rarely increased eosinophils [16]. Using only one eosinophil measurement in patients with AECOPD, a retrospective study of 811 patients also showed that eosinophilic exacerbations (≥ 300 cells/µl) had a lower three-year mortality [20] and similar results were obtained when other threshold values (≥ 2% of total WBC or ≥ 150 cells/µl) were used for the eosinophilic phenotype [19, 29].

The possible reasons underlying the association between BEC and risk of exacerbation or death are not clearly understood. In our study, patients in the LL group were older and had a higher CRP level, which might be related to the relatively higher mortality. In fact, Russell et al. have found that low levels of blood eosinophils were a poor prognostic sign and CRP was elevated in those who died [30]. Moreover, non-eosinophilic AECOPD had a higher risk of in-hospital mortality [10]. In addition, lower BEC was associated with a higher treatment failure rate because of a poorer response to systemic corticosteroid treatment [31]. The increased risk of exacerbation in the HH group might be due to an inadequate treatment course or a lack of maintenance treatment. Researchers found that patients were more likely to be discharged when they had a slightly lower neutrophil count [30]. A better response to corticosteroid therapy in patients of group HH might lead them to discontinue medications prematurely. The potential differences in prognosis and complicated mechanisms need to be further studied.

The present study had several limitations. First, it was a single-center study and the results may not be generalizable to the entire patients hospitalized for AECOPD. Second, it was impossible to control patients in the four groups admitted at similar periods of their exacerbation because of lack of data regarding when their symptoms started, which potentially confounded clinical outcomes. However, its “real-life” nature without any intervention reflected the realities of AECOPD patients admitted in the tertiary hospital and provided valuable information to clinical management of patients requiring hospitalization. Such data collection will be planned in our future related studies in order to better understand the role of blood eosinophils in patients with AECOPD. Third, our findings have limited external validity to females with AECOPD due to the small number of females recruited. Fourth, the uneven distribution of four groups and the small number of deaths during follow-up limited the ability to find the potential significant associations. Fifth, not all patients included in this study had follow-up data within 12 months, but the clinical characteristics showed no significant difference between patients with or without follow-up data, making selective bias less likely. Finally, the confounders from unmeasured variables, such as antibiotic use during admission and medication treatment during follow-up, could also influence the results.

## Conclusions

This study found that in hospitalized AECOPD, more than one-third of patients admitted with a BEC ≥ 300 cells/µl were discharged with a BEC < 300 cells/μl. Patients whose eosinophil counts were low at admission but high at discharge had a poor short-term prognosis. A persistently high BEC during admission was associated with a higher risk of future exacerbations. At least two eosinophil counts seem to be necessary for identifying patients with different clinical characteristics.

## Supplementary Information

**Additional file 1: Table S1.** Clinical characteristics of the patients with or without follow-up data. **Table S2.** Clinical characteristics of patients without asthma and without use of systemic corticosteroids prior to admission. **Table S3.** Cox hazard analyses in patients without asthma and use of systemic corticosteroids prior to admission.

## Data Availability

The datasets used and/or analyzed during the current study are available from the corresponding author on reasonable request.

## Abbreviations

COPD: Chronic obstructive pulmonary disease
RCTs: Randomized controlled trials
AECOPD: Acute exacerbation of COPD
BEC: Blood eosinophil count
ICU: Intensive care unit
GOLD: Global initiative for chronic obstructive lung disease
ICS: Inhaled corticosteroids
mMRC: Modified Medical Research Council
CAT: COPD Assessment Test
CRP: C-reactive protein
IQR: Interquartile range
SD: Standard deviation
WBC: White blood cells
IMV: Invasive mechanical ventilation
FEV_1_: Forced expiratory volume in 1 s
FVC: Forced vital capacity
LAMA: Long-acting muscarinic receptor antagonist
LABA: Long-acting beta-adrenoceptor agonist
NIV: Noninvasive ventilation
