# Supplementary material for: Stability of blood eosinophils in acute exacerbation of chronic obstructive pulmonary disease and its relationship to clinical outcomes: a prospective cohort study
Source: Respir Res. 2021 Nov 24;22:301. doi: 10.1186/s12931-021-01888-5 (PMC8611944; doi:10.1186/s12931-021-01888-5)
Supplement: Supplementary file 1 — Additional file 1: Table S1. Clinical characteristics of the patients with or without follow-up data. Table S2. Clinical characteristics of patients without asthma and without use of systemic corticosteroids prior to admission. Table S3. Cox hazard analyses in patients without asthma and use of systemic corticosteroids prior to admission. [file 12931_2021_1888_MOESM1_ESM.docx]

**Table S1 Clinical characteristics of the patients with or without follow-up data**

| **Variables** | **With follow-up data (n=349)** | **Without follow-up data (n=181)** | ***P*-value** |
| --- | --- | --- | --- |
| Age (years) | 67 (63-76) | 70 (65-76) | 0.216 |
| Male | 320 (91.7%) | 163 (90.1%) | 0.629 |
| Body-mass index (kg/m^2^) | 21.3 (18.7-23.9) | 20.9 (18.5-24.4) | 0.950 |
| Smoking status^a^ |  |  | 0.094 |
| Non-smoker | 38 (11.0%) | 32 (17.7%) |  |
| Ex-smoker | 203 (58.8%) | 101 (55.8%) |  |
| Current smoker | 104 (30.1%) | 48 (26.5%) |  |
| Post-bronchodilator FEV_1_% predicted | 32.0 (22.3-43.1) | 33.9 (24.5-43.0) | 0.400 |
| Post-bronchodilator FEV_1_/FVC (%) | 36.9 (30.3-49.0) | 39.3 (32.0-47.1) | 0.336 |
| 6-Min walk test (m) | 150.0 (10.0-306.0) | 100.0 (10.0-264.0) | 0.489 |
| mMRC | 3 (3-4) | 3 (2-4) | 0.810 |
| CAT | 24 (19-28) | 25 (18-29) | 0.741 |
| Exacerbations in the previous year | 2 (1-3) | 2 (1-3) | 0.782 |
| Pre-admission medication^b^ |  |  |  |
| LAMA | 167 (47.9%) | 71 (40.3%) | 0.115 |
| LABA | 213 (61.0%) | 95 (54.0%) | 0.133 |
| ICS | 209 (59.9%) | 93 (52.8%) | 0.135 |
| Systemic corticosteroids | 32 (9.2%) | 22 (12.5%) | 0.286 |
| Co-morbidity |  |  |  |
| Pneumonia | 63 (18.1%) | 38 (21.0%) | 0.417 |
| Asthma | 31 (8.9%) | 20 (11.0%) | 0.440 |
| Hypertension | 137 (39.3%) | 63 (34.8%) | 0.345 |
| Ischaemic heart disease | 62 (17.8%) | 28 (15.5%) | 0.544 |
| Diabetes | 49 (14.0%) | 23 (12.7%) | 0.691 |
| Cerebrovascular accident | 24 (6.9%) | 12 (6.6%) | 1.000 |
| Respiratory failure | 157 (45.0%) | 75 (41.4%) | 0.461 |
| EOS (10^9^/L) on admission | 0.12 (0.04-0.22) | 0.10 (0.04-0.21) | 0.820 |
| EOS (10^9^/L) on discharge | 0.17 (0.09-0.27) | 0.18 (0.10-0.29) | 0.799 |

Data are presented as n (%) or median (IQR). ^a^Smoking status has 4 missing values. ^b^Pre-admission medication has 5 missing values.

FEV_1_, forced expiratory volume in 1 s; FVC, forced vital capacity; mMRC, modified Medical Research Council; CAT, COPD Assessment Test; LAMA, long-acting muscarinic receptor antagonist; LABA, long-acting beta-adrenoceptor agonist; ICS, inhaled corticosteroids; EOS, eosinophils.

**Table S2 Clinical characteristics of patients** **without asthma and without use of systemic corticosteroids prior to admission**

| **Variables** | **Total cohort (n=434)** | **Blood eosinophil count** | | | | |
| --- | --- | --- | --- | --- | --- | --- |
|  |  | **LL (n=319)** | **LH (n=46)** | **HL (n=22)** | **HH (n=47)** | ***P*-value** |
| Age (years) | 69 (63-76) | 69 (64-77) | 68 (62-76) | 66 (55-71)^a^ | 66 (61-72)^a^ | 0.004 |
| Male | 401 (92.4%) | 299 (93.7%) | 42 (91.3%) | 18 (81.8%) | 42 (89.4%) | 0.139 |
| Body-mass index (kg/m^2^) | 21.1 (18.7-24.0) | 20.9 (18.6-24.2) | 21.9 (19.1-24.0) | 21.5 (20.1-23.3) | 20.3 (17.9-23.3) | 0.835 |
| Smoking status^c^ |  |  |  |  |  | 0.504 |
| Non-smoker | 51 (11.8%) | 33 (10.4%) | 6 (13.0%) | 5 (22.7%) | 7 (14.9%) |  |
| Ex-smoker | 248 (57.5%) | 181 (57.3%) | 28 (60.9%) | 10 (45.5%) | 29 (61.7%) |  |
| Current smoker | 132 (30.6%) | 102 (32.3%) | 12 (26.1%) | 7 (31.8%) | 11 (23.4%) |  |
| Post-bronchodilator FEV_1_% predicted | 32.0 (22.4-43.0) | 32.2 (23.0-43.0) | 31.0 (19.0-48.0) | 33.0 (22.6-37.0) | 30.5 (22.3-39.8) | 0.788 |
| Post-bronchodilator FEV_1_/FVC (%) | 37.7 (31.0-48.0) | 37.8 (31.2-50.0) | 32.2 (27.4-49.0) | 35.3 (31.7-45.0) | 35.4 (32.1-40.9) | 0.254 |
| 6-Min walk test (m) | 136.0 (10.0-305.5) | 129.0 (10.0-309.5) | 100.0 (2.5-249.0) | 189.0 (50.0-294.0) | 208.5 (50.0-360.0) | 0.393 |
| mMRC | 3 (2-4) | 3 (2-4) | 4 (2-4) | 3 (3-4) | 3 (2-4) | 0.346 |
| CAT | 24 (19-29) | 24 (19-28) | 23 (19-30) | 27 (22-30) | 25 (18-30) | 0.513 |
| Exacerbations in the previous year | 2 (1-3) | 2 (1-3) | 2 (1-2) | 2 (1-4) | 2 (1-4) | 0.337 |
| Pre-admission medication^d^ |  |  |  |  |  |  |
| LAMA | 192 (44.5%) | 141 (44.2%) | 21 (48.8%) | 8 (36.4%) | 22 (46.8%) | 0.803 |
| LABA | 242 (56.1%) | 177 (55.5%) | 24 (55.8%) | 11 (50.0%) | 30 (63.8%) | 0.682 |
| ICS | 236 (54.8%) | 174 (54.5%) | 24 (55.8%) | 10 (45.5%) | 28 (59.6%) | 0.747 |
| Co-morbidity |  |  |  |  |  |  |
| Pneumonia | 85 (19.6%) | 63 (19.7%) | 10 (21.7%) | 4 (18.2%) | 8 (17.0%) | 0.944 |
| Hypertension | 162 (37.3%) | 121 (37.9%) | 17 (37.0%) | 8 (36.4%) | 16 (34.0%) | 0.968 |
| Ischaemic heart disease | 72 (16.6%) | 53 (16.6%) | 6 (13.0%) | 2 (9.1%) | 11 (23.4%) | 0.410 |
| Diabetes | 58 (13.4%) | 45 (14.1) | 4 (8.7%) | 4 (18.2%) | 5 (10.6%) | 0.634 |
| Cerebrovascular accident | 31 (7.1%) | 26 (8.2%) | 4 (8.7%) | 1 (4.5%) | 0 (0.0%) | 0.155 |
| Respiratory failure | 195 (44.9%) | 151 (47.3%) | 18 (39.1%) | 7 (31.8%) | 19 (40.4%) | 0.358 |
| CRP (mg/L) on admission | 11.7 (4.0-37.6) | 11.7 (4.4-38.0) | 22.5 (6.5-75.5) | 5.4 (2.7-15.6)^b^ | 7.4 (2.4-20.6)^a, b^ | 0.011 |
| Systemic corticosteroids during admission | 352 (81.1%) | 261 (81.8%) | 35 (76.1%) | 19 (86.4%) | 37 (78.7%) | 0.711 |
| Respiratory support during admission |  |  |  |  |  |  |
| Oxygen therapy | 293 (67.5%) | 215 (67.4%) | 30 (65.2%) | 13 (59.1%) | 35 (74.5%) | 0.608 |
| NIV | 200 (46.1%) | 156 (48.9%) | 22 (47.8%) | 8 (36.4%) | 14 (29.8%) | 0.074 |
| IMV | 4 (0.9%) | 3 (0.9%) | 1 (2.2%) | 0 (0.0%) | 0 (0.0%) | 0.537 |
| ICU during admission | 50 (11.5%) | 39 (12.2%) | 10 (21.7%) | 0 (0.0%)^b^ | 1 (2.1%)^a, b^ | 0.009 |
| Length of stay in hospital (days) | 10 (8-13) | 10 (9-13) | 12 (9-14)^a^ | 11 (8-14) | 10 (8-12)^b^ | 0.003 |
| Total cost during admission (US$) | 2528 (1924-3328) | 2592 (1894-3400) | 2611 (2141-4991)^a^ | 2377 (1895-2854)^b^ | 2176 (1746-2642)^b^ | <0.001 |

Data are presented as n (%) or median (IQR). ^a^Compared with the LL group, P < 0.05. ^b^Compared with the LH group, P < 0.05. ^c^Smoking status has 3 missing values. ^d^Pre-admission medication has 3 missing values.

FEV_1_, forced expiratory volume in 1 s; FVC, forced vital capacity; mMRC, modified Medical Research Council; CAT, COPD Assessment Test; LAMA, long-acting muscarinic receptor antagonist; LABA, long-acting beta-adrenoceptor agonist; ICS, inhaled corticosteroids; CRP, C-reactive protein; NIV, noninvasive ventilation; IMV, invasive mechanical ventilation; ICU, intensive care unit.

**Table S3** **Cox hazard analyses in patients without asthma and use of systemic corticosteroids prior to admission**

|  | **Moderate-to-severe exacerbations** | | **All-cause death** |  |
| --- | --- | --- | --- | --- |
|  | **HR (95% CI)^a^** | ***P*-value** | **HR (95% CI)^a^** | ***P*-value** |
| Blood eosinophil count |  |  |  |  |
| LL | 1 (Ref) |  | 1 (Ref) |  |
| LH | 1.060 (0.660-1.701) | 0.811 | 0.823 (0.290-2.334) | 0.714 |
| HL | 1.517 (0.851-2.706) | 0.158 | 0.457 (0.061-3.424) | 0.446 |
| HH | 1.899 (1.171-3.080) | 0.009 | 0.342 (0.045-2.573) | 0.297 |

^a^Cox proportional hazards model.
